# Reply to Comment on Maani Hessari, N.; van Schalkwyk, M.C.; Thomas, S.; Petticrew, M. Alcohol Industry CSR Organisations: What Can Their Twitter Activity Tell Us about Their Independence and Their Priorities? A Comparative Analysis. *Int. J. Environ. Res. Public Health* 2019, *16*, 892

**DOI:** 10.3390/ijerph16142576

**Published:** 2019-07-18

**Authors:** Nason Maani Hessari, May CI van Schalkwyk, Sian Thomas, Mark Petticrew

**Affiliations:** 1 Department of Health Services Research and Policy, London School of Hygiene and Tropical Medicine; London WC1H 9SH, UK; 2 Department of Primary Care and Public Health, Imperial College London, London W6 6RP, UK; 3 Department of Public Health, Environments and Society, London School of Hygiene and Tropical Medicine, London WC1H 9SH, UK

We are grateful for the opportunity to respond to the letter from the DrinkAware medical advisory panel. It is disappointing that their response fails to acknowledge the findings of our paper: significant differences between industry-funded and non-industry-funded charities in the topics of their communication via Twitter. These differences reflect previously-documented alcohol industry framings and biases. Instead of addressing the findings, their response focuses on defending DrinkAware while attacking the authors, the journal, the peer reviewers, and the review process.

In particular, their statement that “Drinkaware’s ability to reach millions of lay people to educate them in the harms of alcohol is well recognised and its record in using Twitter effectively in this purpose, is unrivalled” is concerning. On the contrary, our analysis (and other evidence) suggests that this “reach” facilitates the effective dissemination of misinformation about alcohol harms. The extent of this dissemination may, indeed, be unrivalled.

The authors also claim that the analysis is a selective criticism of DrinkAware. This is untrue. We analysed the Twitter activity of three national-level, alcohol industry-funded charities and compared them to three national-level, independently-funded alcohol charities and included a statistical analysis of the most commonly mentioned topics. An examination of the data in the paper, however, shows that DrinkAware stands out from other organisations in terms of the topics it focusses on, and those it does not.

The panel was also “disturbed at the objectives” of this study. To reiterate, the objectives of the study were to analyse the social media activities of a number of alcohol-industry-funded and non-AI-funded organisations which disseminate information about alcohol and health to the public. It is therefore misleading to represent this as a study about DrinkAware. However, given previous published, peer-reviewed evidence [1,2,3,4] of the dissemination of misleading health information by AI-funded corporate social responsibility (CSR) organisations, including DrinkAware, it is a legitimate and important focus of research, not least to protect the public from AI-funded misinformation.

We note that the panel states that since DrinkAware is an alcohol education charity, it should not be described as an alcohol-industry corporate social responsibility organisation. Clearly, the status of DrinkAware as a charity is not incompatible with it forming part (arguably a large part, since the DrinkAware logo is on all alcohol industry adverts and labels) of alcohol industry corporate social responsibility activities.

We note also that the panel states that our statement that “the purpose of such alcohol industry CSR organisations is to protect the alcohol market and their reputation” is not based on evidence but on our own beliefs and suggest that their website is proof of this.

In fact, the findings of this paper, as well as previous research, both by us [2,3,5] and others [1], support the growing evidence [6,7] that such bodies are funded by the industry as part of efforts to avoid regulation and to protect sales. The panel’s claim that their website has considerable reach is in itself not an indication of effectiveness. Organisations including the WHO, OECD, and PHE have concluded based on the evidence that industry-funded education campaigns have no meaningful impact on alcohol consumption [8]. That is perhaps why it is favourable for the industry to fund them, while opposing measures with strong evidence of reducing consumption, such as taxation or marketing restrictions [7]. The panel may believe that DrinkAware’s activities contribute to improving people’s health. However, their belief does not reflect the evidence—an evidence base which peer-reviewed research, such as the current paper, as well as previous papers, contributes to.

The panel also cite an earlier letter to the editor they wrote regarding a previous paper which showed how alcohol-industry-funded organisations were misleading the public on cancer. That previous paper, co-authored by some of the authors of the current study, reported three main tactics: (i) denial/omission: denying, omitting or disputing the evidence that alcohol consumption increases cancer risk; (ii) distortion: mentioning cancer but misrepresenting the risk; and (iii) distraction: focussing discussion away from the independent effects of alcohol on common cancers. That particular study analysed data from 27 alcohol-industry-related organisations around the world, including DrinkAware. The panel suggest that as a result of their letter to the editor being published, the evidence in that paper is in fact “unsupported assertions”, and that for the sake of balance, we should have noted this in this most recent article. This is an unusual understanding of the scientific process, apparently suggesting that a peer-reviewed publication based on systematic data collection and analysis is merely “assertion”, whereas their letter to the editor, is “evidence”. Furthermore, in their call for balance, the panel have omitted to note that we in turn wrote a response at the time rebutting their criticisms (3), which pointed them to further evidence of misleading information on cancer from DrinkAware. The earlier analysis remains as important evidence of such activities, and has been subsequently cited as such by other researchers in the field. 

It is implied in their response that this previous paper is somehow the sole evidence that alcohol-industry funding might bias an organisation’s output, or that such funding is made with a view to protecting the profits of the alcohol industry. This is clearly not the case. Such organisations, including DrinkAware, have been the subject of other analyses [1,9,10] and that there is a growing evidence base regarding the wide-ranging activities of the alcohol industry in resisting evidence-based policies that might impact on profit while funding ineffective approaches [6,11,12,13,14]. Moreover, there is an abundant literature regarding the links between industry funding and bias [15]. To suggest that industry funding does not induce some form of positive bias in general is clearly unsupported by the weight of evidence.

In conclusion, we have presented evidence of alcohol-industry-friendly bias in alcohol-industry-funded charity materials, a finding which builds on previous evidence. The obvious unanswered question now is—at what point in the process of developing such materials for public dissemination is this industry-friendly and misleading messaging introduced, by whom, and how?
